# Genes encoding pentatricopeptide repeat (PPR) proteins are not conserved in location in plant genomes and may be subject to diversifying selection

**DOI:** 10.1186/1471-2164-8-130

**Published:** 2007-05-23

**Authors:** Rachel Geddy, Gregory G Brown

**Affiliations:** 1 Plant Products Directorate, Plant Biosafety Office, Canadian Food Inspection Agency, 59 Camelot Drive, Ottawa, Ontario, K1A 0Y9, Canada; 2 Department of Biology, McGill University, Montreal, Quebec, H3A 1B1, Canada

## Abstract

**Background:**

The pentatricopeptide repeat (PPR) is a degenerate 35 amino acid motif that occurs in multiple tandem copies in members of a recently recognized eukaryotic gene family. Most analyzed eukaryotic genomes contain only a small number of PPR genes, but in plants the family is greatly expanded. The factors that underlie the expansion of this gene family in plants are not as yet understood.

**Results:**

We show that the location of PPR genes is highly variable in comparisons between orthologous, closely related, and otherwise co-linear chromosomal regions of the *Brassica rapa *or radish and *Arabidopsis thaliana*. This observation also pertains to paralogous duplicated segments of the genomes of *Arabidopsis thaliana *and *Brassica rapa*. In addition, we show that PPR genes that seem closely linearly aligned in these comparisons are not generally found to be closely related to one another at the nucleotide and amino acid sequence level. We observe a relatively high level of non-synonomous vs synonomous changes among a group tandemly repeated radish PPR genes, suggesting that these, and possibly other PPR genes, are subject to diversifying selection. We also show that a duplicated region of the *Arabidopsis *genome possesses a relatively high density of PPR genes showing high similarity to restorers of fertility of cytoplasmic male sterile (CMS) systems of petunia, radish and rice. The PPR genes in these regions, together with the restorer genes, are more highly similar to one another, in sequence as well as in structure, than to other PPR genes, even within the same sub-family.

**Conclusion:**

Our results suggest are consistent with a model in which at least some PPR genes undergo a "birth and death" process that involves transposition to unrelated chromosomal sites. PPR genes hold certain features in common with disease resistance genes (R genes), and their "nomadic" character suggests that their evolutionary expansion in plants may have involved novel molecular processes and selective pressures.

## Background

The pentatricopeptide repeat (PPR) peptide motif, first described by Small and Peeters [1], is a degenerate 35 amino acid sequence, closely related to the 34 amino acid tetratricopeptide repeat (TPR) motif. TPRs occur as tandem repeats in a widespread protein family of both prokaryotes and eukaryotes. PPRs also occur in multiple tandem repeats, but have thus far been found to be exclusively eukaryotic in their distribution. On the basis of the solved structure of a TPR domain [2] as well as modelling approaches [1], each PPR domain is though to be configured as two distinct anti-parallel alpha-helices, helices A and B. In PPR proteins, tandem repeats of these alpha-helical pairs are predicted to form a superhelix that encloses a central spiral groove with a positively charged ligand-binding surface [1]. PPR proteins are known to mediate specific RNA processing events including RNA editing [3], transcript processing [4], and translation initiation [5], and are thus thought to be capable of specific binding to both protein and RNA molecules.

Although all sequenced eukaryotic genomes have been found to encode PPR proteins, the numbers of PPR genes in both animal and fungal genomes is relatively small. In plants, however, the size of this gene family is greatly expanded. In *Arabidopsis thaliana *there are 441 identified PPR genes and more than 655 PPR proteins have been predicted to occur in the rice genome [6].

Analysis of the PPR gene content of the *Arabidopsis *genome by Lurin *et al*. [6] elucidated several categories and subcategories of PPR genes. The largest category encodes proteins that are composed of tandem repeats of the "classical" 35 amino acid PPR motif initially described by Small and Peeters [1], and now referred to as the P-type repeat. Lurin *et al*. [6] were able to differentiate three additional PPR-related motifs found in PPR-encoding genes. Two of these motifs, S and L1, are tandemly arrayed with the classical P-type motif in a repeated P-L1-S (PLS) pattern, with the third motif, L2, replacing L1 in the last repeat pattern at the C-terminal end of the protein. Their analyses also showed that the PLS subfamily of PPR-encoding genes is unique to plants and not found in other systems. Four subgroups of PPR proteins from the PLS subfamily differ in the structure of their C-terminal domains. Although two of the subgroups, E and E+, are highly degenerate in their C-terminal sequences, the DYW subgroup shows some conservation of amino acid residues. It has been suggested that this C-terminal domain may function as a catalytic domain for these PPR proteins [6]. One PPR gene belonging to the PLS subfamily is *Emb175*, a gene essential for plant embryogenesis. *EMB175*, like many PPR proteins, is targeted to the plastid [7].

Another major group of plant-specific PPR genes are the restorer of fertility (Rf) genes. These nuclear-encoded genes act to suppress male sterility associated with cytoplasmic male sterility (CMS), a phenomenon related to the expression of mitochondrially-encoded sterility-associated genes. Rf genes identified thus far in petunia, rice and radish belong to the P subfamily of PPR genes [8-10].

Expansion of the complement of PPR genes within plant genomes may have occurred through gene duplication. In *Arabidopsis*, ancient large-scale genome duplication events have resulted in multiplication of loci and regions of synteny, where gene number and location are conserved as paralogous copies. Gene duplication can also arise from tandem and segmental gene duplication, creating clusters of identical genes that diverge over time [11,12]. With the exception of one location on chromosome 1, no clustering of PPR genes has been reported for *Arabidopsis *[6]. However, tandem clusters of PPR genes have been observed in petunia [8] radish [10] and rice [9,13,14].

The synteny observed in duplicated genomic regions within a genome or between orthologous copies in related genomes can be exploited in gene mapping. However, disruptions of synteny can occur due to gene loss, rearrangement, acquisition or duplication. The source of these structural changes can be due to tandem and segmental duplication, as discussed above, but could also be attributed to aberrant homologous recombination, selection, or changes introduced by transposition events.

The radish restorer of fertility, *Rfo*, is found in a cluster of PPR genes at a genomic site where no corresponding PPR gene is found in the syntenic region of *Arabidopsis *[10]. We report here that other PPR genes display a characteristic lack of synteny in comparisons of both orthologous and paralogous plant genomic regions. We show that while non-PPR genes are largely co-linear in arrangement and identical in orientation between different related regions, PPR genes are rarely maintained in the same position or orientation when two related regions are compared. We show that PPR gene family members share characteristics with plant disease resistance genes (R genes); in particular we present evidence that at least some PPR genes, as per R genes, are subject to diversifying selection, i.e. an evolutionary process that selects for, rather than against, mutations that lead to amino acid replacements in the encoded proteins. Such diversifying selection processes may also act to multiply and distribute copies of the genes. Our results also suggest that the Birth-and-Death process initially described for immunoglobulin genes [15], and adapted by Michelmore and Meyers [16] for R genes, may apply as well to the duplication and divergence of PPR genes.

## Results

### Locations of PPR genes are highly variable between co-linear regions of Arabidopsis and Brassica or Raphanus genomes

**Figure 1:**
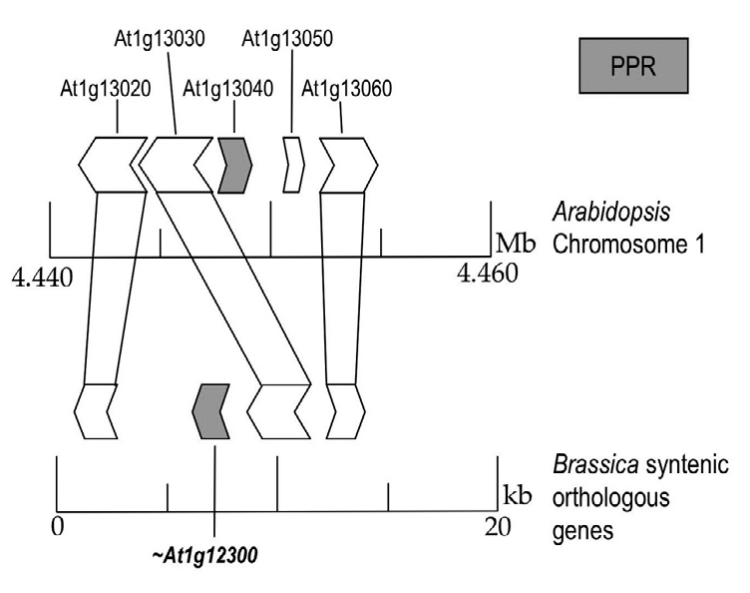
**Predicted genes in *Brassica *and the syntenic segment of the *Arabidopsis *genome**. Protein-coding sequences in the region of the *Brassica *genome represented in clone P2 (bottom) as predicted by Genscan. Dark filled arrows indicate PPR-encoding sequences. Protein-coding sequences in the *Arabidopsis *genome reflect the Munich Information Center for Protein Sequences (MIPS) annotation. The direction of the arrows indicates the direction of transcription/translation. The connecting lines indicate orthologous coding regions in the two genomic sequences. Distances in megabasepairs in *Arabidopsis *are indicative of the location of sequences on chromosome 1. The PPR encoding gene in *Brassica *is asyntenic with respect to the *Arabidopsis *sequences shown here. In addition, it shares homology with *Arabidopsis *At1g12300, and not with its most closely linearly aligned partner At1g13040.

We have sequenced *Brassica *genomic regions in an effort to identify and characterize the fertility restorer gene *Rfp *of *Brassica napus*. The sequences were isolated from *Brassica rapa *genomic DNA introgressed with *Rfp*. One such region, represented by cosmid clone P2, contained four predicted protein-coding genes, one of which could potentially specify a protein with nine PPR domains. Comparison with the *Arabidopsis *genome sequences revealed a related region on chromosome one, spanning five predicted genes, At1g13020 through At1g13060 (Figure 1). Three of these genes, At1g13020, At1g13030 and At1g13060 are co-linear in arrangement and identical in orientation with their *Brassica rapa *genome counterparts, indicating that synteny is preserved between these chromosomal regions of the two species. A high degree of sequence similarity/identity (47–76%/47–78%) is observed between the proteins encoded by these *Arabidopsis *genes and their *Brassica rapa *counterparts. As is commonly observed in genomic comparisons between *Arabidopsis *and *Brassica*, one of the *Arabidopsis *genes, At1g13050, has no apparent counterpart in the *Brassica rapa *sequence [17-19]. Most segments of the *Arabidopsis *genome are represented at multiple sites in *Brassica *genomes, and the resulting high level of genetic redundancy in *Brassica *may lead to the loss of coding sequences at one or more such sites.

The syntenic region of the *Arabidopsis *genome also contains a predicted gene (At1g13040) that could potentially specify a protein with six PPR domains. In contrast with At1g13020, At1g13030 and At1g13060, this protein, has little similarity with PPR protein encoded by the PPR gene in the co-linear *Brassica rapa *region (26% identity [I], 46% similarity [+]). Moreover, its location, between At1g13030 and At1g13050, is different from that of the *Brassica rapa *PPR gene, which is positioned between orthologs of At1g13020 and At1g13030; its transcriptional orientation, with respect to the co-linear genes of the region, also differs. Interestingly, this *Brassica *PPR protein does show a high degree of sequence similarity with *Arabidopsis *PPR genes present at distinct sites on chromosome one. In particular, it possesses 69% identity and 81% similarity with the protein encoded by At1g12300, a PPR gene located in a cluster of such genes near the 4.3 megabase (Mb) mark of chromosome one. Thus, we observe a preservation of synteny for most genes between these *Arabidopsis *and *Brassica *genomic regions but an apparent lack of synteny for the PPR genes. This suggests that the function and order of the non-PPR genes in the region has been conserved during the evolution of the Brassicaceae, but that one, and possibly both of the PPR genes in these two related chromosome regions has descended from a progenitor located at a distinct, non-syntenic chromosomal site.

**Figure 2:**
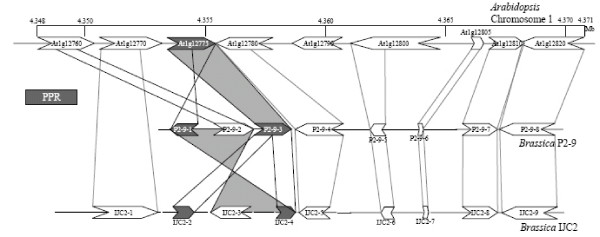
**Predicted genes in two regions of *Brassica napus *and the syntenic segment of the *Arabidopsis *genome**. Protein-coding sequences in the *Brassica *genome fragments P2-9 and IJC2 (as predicted by Genscan). For clarity, *Brassica *predicted genes are numbered. Protein-coding sequences in the *Arabidopsis *genome reflect the Munich Information Centre for Protein Sequences (MIPS) annotation. The direction of arrows indicates the direction of transcription/translation. Pairs of connecting lines indicate orthologous coding sequences between *Arabidopsis *and *Brassica*; darker pairs of lines connect orthologous PPR encoding sequences and other important sequences. Shading between connecting lines is added for emphasis and clarity. Dark-filled arrows indicate PPR encoding sequences. *Brassica *sequences shown are limited by the size of fragment sequenced, thus genes that are outside these sequences are not available.

The lack of synteny with respect to related PPR genes was evident in additional comparisons of *Brassica rapa *and *Arabidopsis *genomic sequences. Two cosmids, P2-9 and IJC2, containing *Brassica rapa *DNA sequences encoding PPR domains were sequenced. P2-9 and IJC2 are paralogous regions that show high structural similarity to one another (Figure 2). P2-9 contains eight predicted protein coding genes and IJC2 contains nine. In both cases, two of the genes encode PPR domains (P2-9-1, P2-9-3, IJC2-2 and IJC2-4). These PPR gene sequences are 80–90% similar with areas of higher similarity within exons. Between the two *Brassica rapa *sequences, gene order and direction of transcription is conserved between the two cosmids except in the region surrounding PPR genes.

IJC2 and P2-9 both show extensive similarity to a region of *Arabidopsis *chromosome I spanning the nine genes flanked by At1g12760 and At1g12820 (Figure 2); one of these *Arabidopsis *genes, At1g12775, encodes a PPR domain protein. The nucleotide sequence identity between the *Arabidopsis *and *Brassica *coding sequences in this region is 85–90%. Gene order is maintained between *Arabidopsis *and the two *Brassica rapa *sequences, with a few exceptions. No counterpart of At1g12790, and only a portion of At1g12800, are found in the *Brassica *cosmids.

The *Brassica rapa *gene P2-9-3 is similar in both sequence and orientation to the *Arabidopsis *PPR gene At1g12775. P2-9-1 is a duplication of the 3' end of P2-9-3. In addition, P2-9-1 and P2-9-2 are represented in IJC2 as IJC2-4 and IJC2-3, respectively, and are inverted in orientation with respect to their P2-9 counterparts. It is likely that after the genomic duplication leading to the formation of these paralogous regions, a local rearrangement occurred. This rearrangement may have excised the P2-9-1/P2-9-2//IJC2-4/IJC2-3 fragment and reinserted it into the genome, knocking out the 3' end of IJC2-2 and replacing it with the inverted fragment. This may have occurred through the homologous recombination of P2-9-1 and the 3' end of P2-9-3//IJC2-2.

The *Brassica rapa *PPR-encoding open reading frame (ORF) P2-9-1 is found in a genomic region that corresponds to sequences flanking At1g12760; no PPR domain-encoding regions occur in this location in the *Arabidopsis *sequence. As explained above, the duplication of the *Brassica rapa *ortholog of At1g12775, P2-9-3, likely resulted in the presence of multiple PPR sequences. The positioning of P2-9-1 to the left of the At1g12760 ortholog P2-9-2 suggests that a genome rearrangement occurred at this location after the split between *Arabidopsis *and *Brassica *which resulted in the movement of At1g12760/P2-9-2 sequences. Thus, as in other genomic comparisons, PPR encoding regions in P2-9, IJC2 and the corresponding *Arabidopsis *chromosome I segment are more highly rearranged than flanking regions encoding other types of proteins, perhaps as a direct result of the movement of PPR genes.

**Figure 3:**
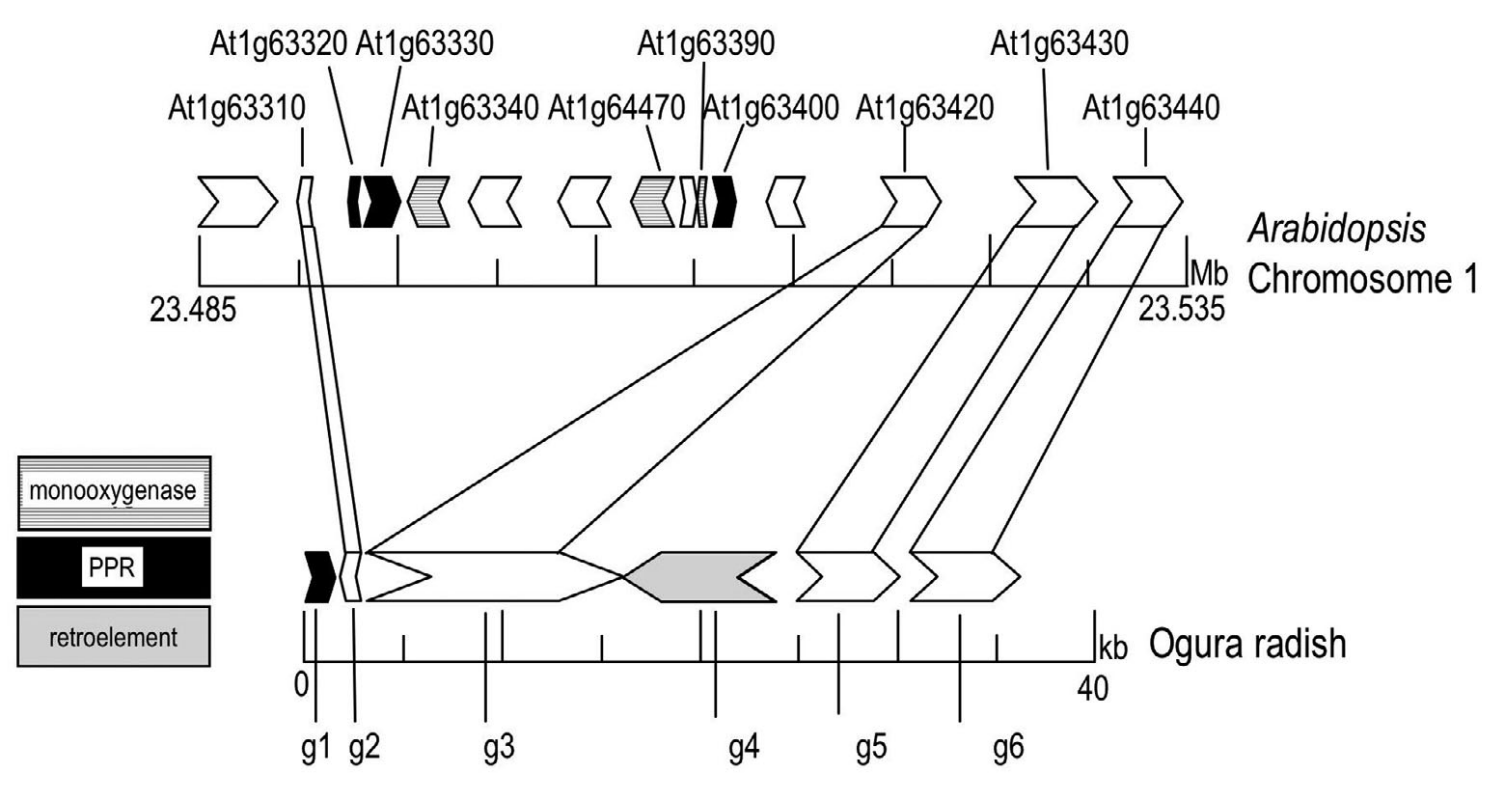
**Predicted genes in radish and the syntenic segment of the *Arabidopsis *genome**. Protein-coding sequences in the radish genome (bottom; as predicted by Genscan). Protein-coding sequences in the *Arabidopsis *genome reflect the Munich Information Center for Protein Sequences (MIPS) annotation. The direction of the arrows indicates the direction of transcription/translation. Dark filled arrows indicate PPR encoding sequences. Arrows with horizontal stripes indicated monooxygenase encoding genes. The gene g4, indicated with a grey filled arrow, encodes a non-LTR retroelement reverse transcriptase. Orthologous coding sequences in the two genomic sequences are indicated by connecting lines. Distances in megabasepairs in *Arabidopsis *are indicative of the location of sequences on chromosome 1.

The segment of the radish (*Raphanus sativum*) genome encoding the *Rfo *restorer gene has been shown to possess extensive co-linearity with the *Arabidopsis *genome [10]. One of the radish genes close to this region, g1, encodes a protein with PPR motifs (Figure 3). The syntenic non-PPR encoding genes of *Arabidopsis *and radish in this region show a high degree of amino acid sequence identity/similarity (63–88%I/67–92%+). Radish g1, however, does not show synteny with *Arabidopsis*, and is located on the opposite side of g2/At1g63310 from its *Arabidopsis *counterparts At1g63320, At1g63330 and At1g63400 with which it is most closely linearly aligned. Although g1 shares similarity to these three PPR genes (35–40%I/55–60%+), it is more similar to *Rfo *(67%I/75%+) than to any *Arabidopsis *gene. Other highly related matches to g1 include the restorers of fertility *Rf1 *in rice (32%I/55%+) and *Rf-1 *in petunia (36%I/57%+). A difference in sequence exists between radish and *Arabidopsis *in this region; a stretch of DNA flanked by PPR genes, and also by three flavin-containing monooxygenase-related genes, is present in *Arabidopsis *but absent in radish (Figure 3). It is possible that a duplication of this fragment present elsewhere in the radish genome rendered the sequences it contains redundant, allowing for excision of this non-essential DNA. The presence of PPR genes flanking the monooxygenase genes may be of significance in the excision of this region from radish, although the mechanism responsible for this change remains unknown at present.

### Variation in PPR gene location between two paralogous gene regions on chromosome I of Arabidopsis

Several restorer genes from various plant species have thus far shown homology to a cluster of PPR genes found in the *Arabidopsis thaliana *genome [6]. This particular set of PPR genes is the largest grouping in the *Arabidopsis *genome of highly homologous PPR genes. This genome segment is located at about the 23 Mb mark of chromosome 1 and includes loci At1g62260 through At1g63630, encompassing 18 PPR genes and pseudogenes [6]. *Rfp*, the restorer of fertility for polima CMS of *Brassica napus*, has been mapped to a genomic region that is syntenic to a portion of the *Arabidopsis *genome located near the 4.3 Mb coordinate of chromosome 1 [20].

**Figure 4:**
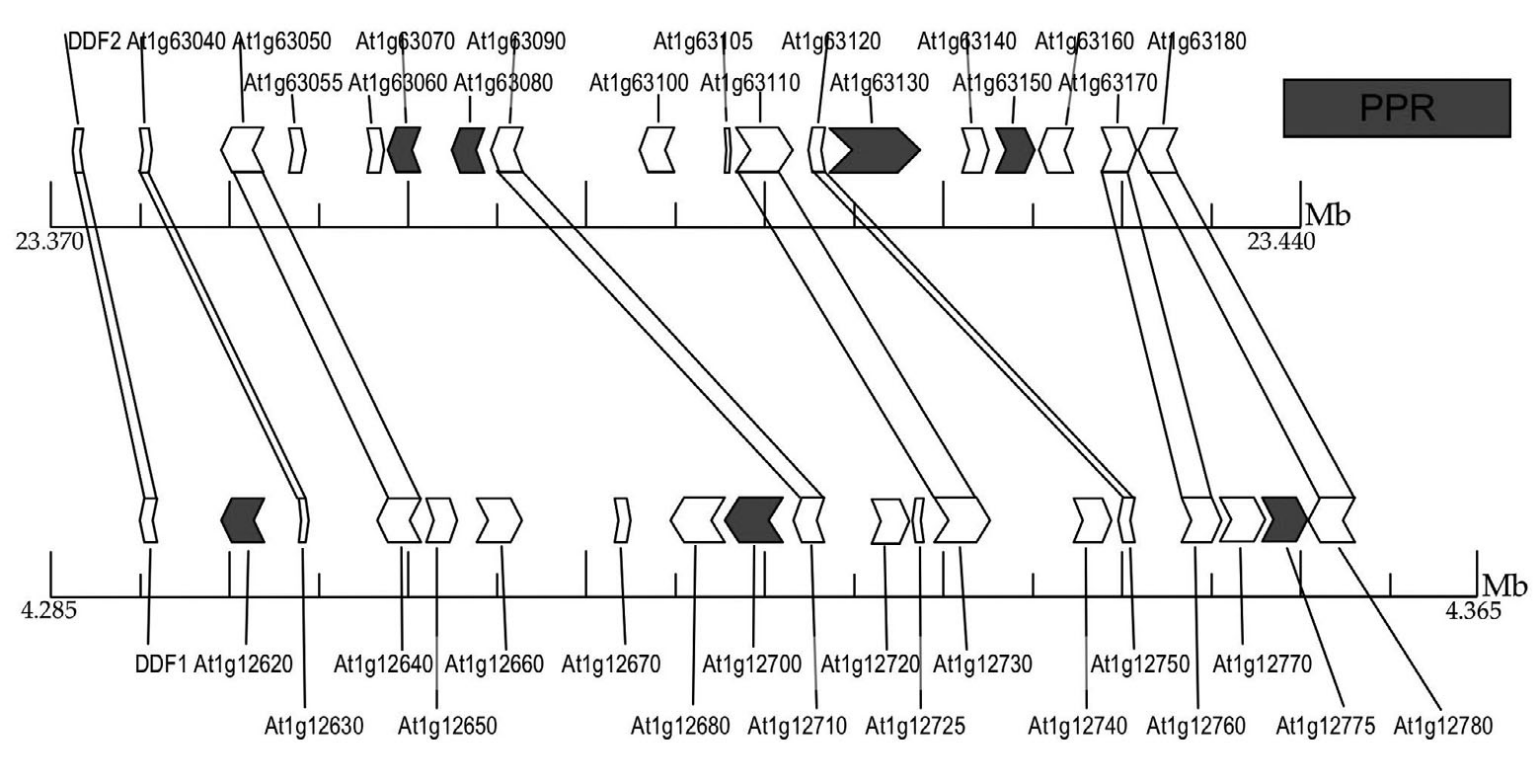
**Comparison of two *Arabidopsis *duplicated regions on chromosome 1**. Protein-coding sequences in the *Arabidopsis *genome chromosome 1 reflect the Munich Information Center for Protein Sequences (MIPS) annotation. The direction of the arrows indicates the direction of transcription/translation. Dark filled arrows indicate PPR encoding sequences. Paralogous coding sequences in the two genomic regions are indicated by pairs of connecting lines. Distances in megabasepairs are indicative of the location of sequences on chromosome.

Previous studies of whole genome internal duplications of the *Arabidopsis *genome have not noted any homology between the 23 Mb and 4.3 Mb regions [21,22]. However, when the *Arabidopsis *genome regions surrounding the 4.3 and 23 Mb coordinates are closely examined, it becomes evident that there is a group of genes that are conserved between these two segments (Figure 4). Recently, this duplication was noted on the website of The Institute for Genomic Research (TIGR) [23]. In the 23.370–23.440 Mb region, there are 18 genes identified by the Munich Information Centre for Protein Sequences (MIPS), comprising four PPR genes, one gene encoding a hypothetical protein and 13 other genes. In the 4.285–4.365 Mb region there are 19 genes, including three PPR genes, two genes encoding hypothetical proteins and a transposase-encoding gene. The two regions share eight predicted coding sequences displaying significant sequence similarity; 50% of the non-PPR encoding genes in the 4.3 Mb region possess a paralogous counterpart in the 23 Mb region. In the 23Mb region 57% of non-PPR encoding genes share a paralogous counterpart in the 4.3 Mb region (Tables 1, 2). Seven of the eight conserved genes are maintained in the same order and transcriptional direction between the two fragments.

**Figure 5:**
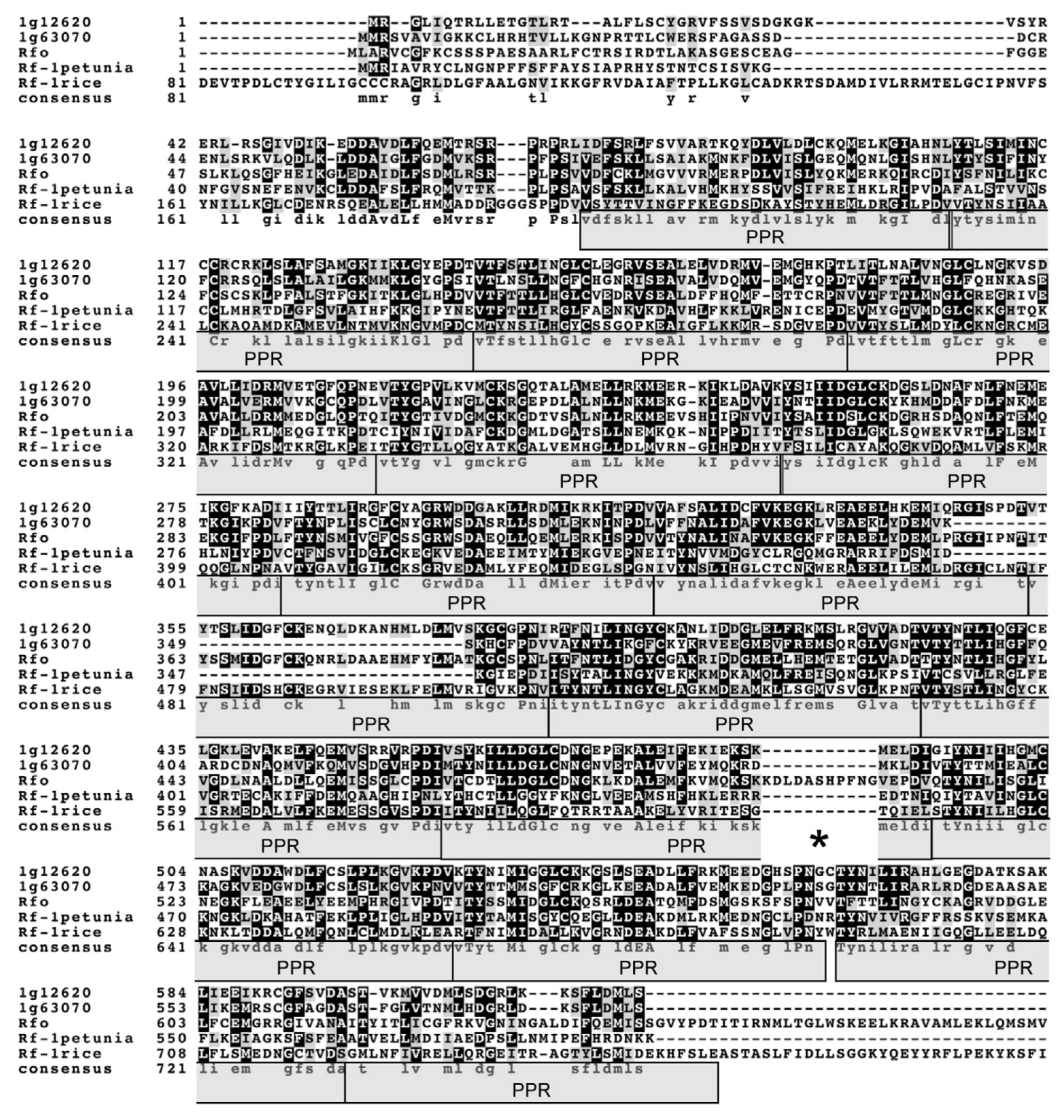
**Alignment of protein coding sequences of PPR encoding restorer of fertility genes with PPR genes of the 4.3 and 23 megabase regions**. *Rfo *from Ogura radish, *Rf-1 *from petunia and *Rf-1 *from rice are all highly related to the PPR genes from *Arabidopsis thaliana *chromosome one 4.3 and 23 megabase regions. They are less highly related to other PPR genes from other subgroups and locations. It is likely that PPR genes which act as restorers all originated from the same progenitor PPR gene. As this alignment shows, the repeat structure among these genes is conserved from sequence to sequence, even between different species. The asterisk (*) indicates a sequence insertion present only in *Rfo*.

It has already been shown by Lurin *et al*. [6] that PPR-containing genes of the *Arabidopsis *genome can be categorized by their structure and sequence similarity to each other, including their C-terminal domains. The group of PPR genes of the 23 Mb region are included in the P subfamily of PPR proteins and are highly similar to those in the 4.3 Mb region (Table 2). The overall similarity among the restorer genes and these two groups of *Arabidopsis *PPR genes is evident in the linear alignment of the proteins encoded by various restorer genes and representatives of the 23 and 4.3 Mb regions PPRs (Figure 5, additional file 1). In comparisons based on this alignment, the 4.3 Mb region PPR genes At1g12620 and At1g12775 are 82% identical (I) and 90% similar (+) at the amino acid level, whereas they are each only about 58% identical and 74% similar to the PPR gene At1g12700. Within the 23 Mb region PPR proteins At1g63070, At1g63080, At1g63130 and At1g63150 are 68–78% identical and 78–88% similar. The outlying PPR gene, At1g63230, could theoretically encode a protein which is less similar to other PPR proteins in the 23 Mb group (52%I/67%+); however, it has been annotated as false by Lurin *et al*. [6] due to the lack of a "plausible initiation codon". The divergent nonfunctional sequence may have arisen from the loss of a start codon, leading subsequently to lower similarity to related PPR sequences.

The overall degree of similarity between between the restorer genes and the related *Arabidopsis *P subfamily PPR genes of the 23 and 4.3 Mb regions is 49%I/66%+, a much higher percentage than between restorers and unrelated P and PLS subfamily PPR genes. This evidence taken together indicates that PPR-encoding restorer genes originate from the same subset of P subfamily PPR genes; no PPR-encoding restorer genes have yet been shown to originate from any other subtype of PPR gene.

**Figure 6:**
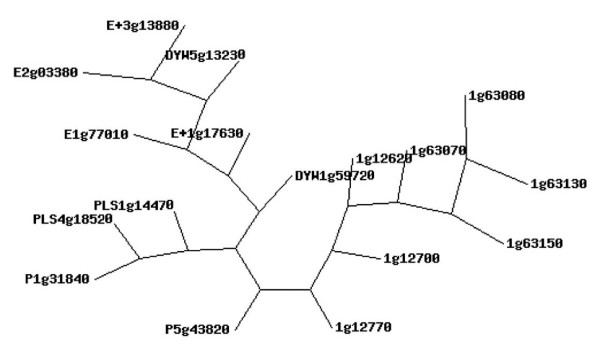
**Phylogenetic relationship between PPR proteins of the 4.3 and 23 Megabase region of *Arabidopsis *chromosome 1 and PPR genes found elsewhere in the *Arabidopsis *genome**. The seven PPR proteins at the right hand side of this unrooted tree are all members of the P subfamily of PPR proteins and belong to the 4.3 or 23 megabase clusters of PPR genes. The other ten PPR proteins are a representative random sampling of PPR proteins of all 5 subfamilies and subgroups (P, E, E+, PLS, DYW, as defined by Lurin *et al*., 2004) from chromosome 1 and another chromosome of *Arabidopsis *chosen at random. PPR proteins of the 4.3 and 23 megabase regions are more closely related to each other than they are to other P-subfamily genes or other PPR genes from other sub-groups, and form a distinct branch away from these PPR proteins.

The close affinity between PPRs encoded in the two *Arabidopsis *regions with respect to other *Arabidopsis *PPRs, including those of the PLS subfamily PPR proteins (ie. E, E+, PLS and DYW subgroups) is also evident in the tree diagram of Figure 6. When At1g12700 (4.3 Mb cluster) is compared with At1g63130 (23 Mb cluster) they are 52% identical and 69% similar. When At1g12700 is compared to PPR proteins from these other subgroups they are only 23% identical and 42% similar. Strikingly, when At1g12700 is compared to other PPR genes of the P subfamily found outside the 23 Mb cluster their proteins are only about 25% identical and 48% similar, a percentage that is not significantly different from the comparison to other PLS subfamily genes. Thus, the PPR proteins found in the duplicated regions of 4.3 Mb and 23 Mb of chromosome 1 are more similar to each other than they are to other PPR proteins, even within the same structural subfamily as described by Lurin *et al*. [6]. The subcellular targeting of PPR genes, however, seems to be independent of subfamily or subgroup of PPR protein (Table 3). It should be noted however, that comparisons of widely diverged repeat family proteins can be problematic [24]. Thus, while the tree in Figure 6 supports the contention that the 23 and 4.3 Mb PPRs are more similar to one another than to other *Arabidopsis *PPRs, no other inference regarding the relationships among the compared proteins should be inferred from this diagram.

As can be seen in Figure 4, the order of genes, and their direction of transcription, is conserved for the non-PPR encoding genes. PPR genes, however, appear to be distributed randomly throughout the two regions. Comparisons of the PPR sequences of the two regions do not reveal a significant correlation of homology between closely linearly aligned PPR genes. For example, At1g12700 shares 50% homology and 65% identity with At1g63070 and At1g63080, with which it is most closely linearly aligned, but this is about the same degree of similarity as is found between it and the other PPR genes in the 23 Mb region.

### PPR genes in the radish Rfo region have been subject to diversifying selection

Clustering of PPR genes, such as that seen in the 23 Mb region of chromosome 1 of *Arabidopsis *is a phenomenon also found associated with disease resistance genes (R genes). R genes are subject to diversifying selection that acts on them in a manner that causes duplication and sequence divergence of genes, thus promoting the creation of new or different genes to combat new pathogens [25]. Brown *et al*. [10] have shown that the PPR-encoding restorer gene for Ogura CMS, *Rfo*, forms a mini-cluster of PPR genes in radish with the genes g24 and g27. These three PPR genes are subject to diversifying selection with a rate of non-synonymous nucleotide substitution (Ka) greater than that of synonymous nucleotide substitution (Ks, Table 4). Conversely, other non-PPR encoding genes of the same region are under the influence of purifying selection with a greater rate of synonymous nucleotide substitution (Table 4). This suggests that PPR genes are under pressure to alter their sequences, thus creating changes that will diversify the population of PPR genes as a whole. This differs from other genes which have a tendency to select against mutation and thus to conserve the sequence of functional proteins. This evidence may lend credence to the hypothesis that PPR genes act as sequence-specific binding proteins, requiring changes in their own sequence to match the sequences they will bind.

## Discussion

Pentatricopeptide repeats (PPR) are structural motifs encoded by a large number of genes in plants and other organisms, although the PPR gene family is greatly expanded in plants. It was hypothesized that this could be due to novel functions served by PPR proteins in plants that are not required in other organisms, or that PPR proteins replace functions performed by other genes in other organisms [6]. Recent evidence shows that PPR proteins can function in chloroplast RNA editing via post-transcriptional conversion of cytosines to uracil [3], supporting the first hypothesis.

Restoration of male fertility is a plant-specific function encoded by PPR genes. Several recently identified restorers of male fertility in plants encode PPRs that are related to each other at the amino acid level. *Rf1 *of petunia [8], *Rf-1 *from rice [9], and *Rfo *from radish [10] are restorers of fertility encoding pentatricopeptide repeat proteins that share approximately 50% amino acid similarity to one another. Since the PPR-encoding restorer genes discovered thus far share sequence similarity, and arise in related gene regions (as is the case with *Rfo *from radish [26]), it seems reasonable to speculate that these genes have arisen from a small number, perhaps even a single, progenitor PPR gene or genes. It is possible that sequences similar to a restorer gene progenitor are located in the 23 Mb region of chromosome I, and that a progenitor of one these genes may have functioned as a restorer gene at some point in the evolutionary past.

We have found that through comparisons of closely related orthologous sequences as well as comparisons of paralogous regions within the *Arabidopsis *and *Brassica *genomes, the locations of genes encoding PPR domain proteins are highly variable relative to the locations of other types of genes. A consideration of the most abundant type of plant disease resistance genes (R genes), NBS-LRR genes, may be useful for understanding the mechanisms underlying PPR gene diversity and evolution. PPR genes and NBS-LRR type R genes share several features in common. Both types of genes encode proteins with a variable number of repetitive motifs, leucine-rich repeats (LRRs) in the case of NBS-LRR type R genes. In both cases, a single dominant gene determines the phenotype, and, in addition, it is the sequence variability within the repeats that lends specificity of action [10,16,27,28]. Finally, the genomic positions of many R genes are not conserved in otherwise syntenic regions of grass genomes [29], similar to the variability in genomic location of PPR genes shown here.

The evolution and diversity of plant disease resistance genes is a result of tandem and segmental gene duplication, recombination, mutation and natural selection [30]. Two sources of gene duplication include local chromosomal rearrangement and large scale genomic duplications [31]; this is consistent with the conserved synteny model of gene evolution that states that these two mechanisms are the cause of gene distribution and long-distance (ectopic) duplication of genes [25]. However, most gene duplications are within restricted local chromosomal segments. These local events are the most recent duplications and are most evident when they interrupt the colinearity of gene order in duplicated chromosomal fragments [25].

A nonconservative mechanism (i.e. a local change of location) would explain the lack of conservation of synteny we observe for PPR genes within closely related genomic segments. Lurin *et al*. [6] suggest that one or more bursts reverse transposition and reintegration could account for the wide distribution of PPR genes among chromosomes, as well as the paucity of introns in these genes. Our data suggest that if retrotransposition underlies transience in location observed among PPR genes, such events occur relatively frequently. Moreover, our findings suggest that following such a transposition event, the original gene copy would be quickly be lost, as we find no evidence for such remnants of PPR genes in comparisons in regions where a PPR gene exists in one, but not other related genomic regions. It is possible that the "nomadic" character of PPR genes reflects as yet unrecognised mechanism of gene duplication and transposition, and results in deviations in synteny in orthologous and paralogous comparisons.

Paralogous PPR genes such as those found in the syntenic *Arabidopsis *4.3 and 23 Mb regions are likely the result of genomic duplications. Such duplicated regions are thought to diverge because they are physically too distant from one another for to allow for intergenic exchange [12]. Instead, sequence variability and changes in copy number within such regions likely results from interallelic recombination and diversifying selection [12]. Over time, tandem gene duplication can occur as is seen in the radish *Rfo *region. Interestingly, this tandem duplication is not evident in *Arabidopsis *PPR gene distribution. PPR genes are for the most part found as singlets with PPR gene subgroup members evenly distributed in the *Arabidopsis *genome amongst the five chromosomes, whereas R genes are found more often in clusters of related genes [32]. This may be a result of the large diversifying selective pressure exerted on disease resistance loci as plants are continually adapting to new plant pathogens [25].

Previous studies showed only one defined cluster of PPR genes in *Arabidopsis thaliana*; it is the cluster related to restorer genes of rice, petunia and radish [6]. It is possible that this clustering indicates diversifying selection acting on PPR genes from that region as a result of plants adapting to newly emerging sterility inducing genes. The diversifying selective pressure exerted on the mini-cluster of PPR genes at the radish *Rfo *locus is one example of this effect acting on PPR genes and not on other genes of the same region. Again, the PPR genes of the *Rfo *region are out of synteny with the PPR genes of *Arabidopsis*, while the non-PPR encoding gene locations are conserved. Diversifying selection may partly explain why non-PPR genes are not apt to fall out of synteny with their paralogous partners. If diversifying selection is not acting on a gene then any changes in the sequence are less likely to lead to changes in gene structure, location or in the amino acid sequence of the encoded protein; synonymous substitution would outweigh nonsynonymous substitution and there would be little selection for sequence location changes [27,33].

Mitochondrially-encoded CMS genes, as well as associated nuclear restorer genes, arise naturally in plant populations. It has been suggested that the spread of maternally-inherited male sterility in a hermaphroditic plant populations may be advantageous, since female individuals would not need to invest resources into the production of pollen [34]. If the frequency of such a gene were to become sufficiently high, it would create selective pressure for the evolution of a corresponding nuclear restorer gene. This scenario has been termed an "intra-genomic arms race" [35]. It is possible that such selective pressure is responsible for the diversification of at least a portion of the PPR genes in a particular plant genome, such as that in the *Rfo *region of radish. Moreover, the maintenance of CMS genes within the mitochondrial genome would provide selective pressure for the maintenance of corresponding restorers in the nuclear genome. The eventual loss of the CMS gene from the mitochondria would allow loss of the restorer gene from the nucleus. This scenario provides one mechanism for the "Birth-and-Death" of plant PPR genes.

The presence of so-called false PPR genes [6] also follows the Birth-and-Death model adopted by Michelmore and Meyers [16] for R genes, which indicates that following gene duplication due to diversifying selection some members have become redundant; mutations which cause frameshifts or premature stop codons in the coding sequences of these genes have had their function disabled. It has been noted that PPR genes contain, on average, many fewer introns than other *Arabidopsis *genes, thus increasing the likelihood that mutations will affect coding regions [6].

## Conclusion

We show here that PPR genes, at least those in the P subfamily, possess a novel, "nomadic" character in that their positions are highly variable in otherwise co-linear segments of closely related genomes. This suggests that they may be undergoing a "Birth-and-Death" process that would involve either non-conservative transposition or conservative transposition followed by rapid loss of the non-transposed copy of the gene. They resemble, in several respects, another versatile gene family of plants, disease resistance genes. The common features exhibited by both types of genes are consistent with the view that PPR genes may, like R genes function as proteins with malleable binding capacities that can undergo rapid alterations in response to changing selective pressures. Since it appears that most PPR genes in plants function by binding to one or a small number of specific target organelle transcripts, it is possible that changes in organelle genomes drive PPR gene evolution and thus the evolution of this gene family in plants likely reflects the co-evolution of nuclear and organelle genomes. This work suggests that evolution of PPR genes in plants may involve novel molecular mechanisms and illustrates one additional feature of this interesting and enigmatic gene family.

## Methods

### *Brassica rapa *cosmid clones

The *Rfp *gene has been mapped to a region of the *Brassica napus *genome syntenic with *Arabidopsis *genome sequence surrounding the 4.3 Mb coordinate on chromosome 1 [20]. Primers for the amplification of gene sequences in this region were designed using the online software Primer3 [36]. These primers were used to amplify the corresponding sequences from *Brassica napus *cv. Westar total DNA using the polymerase chain reaction (PCR) with annealing temperatures varying depending on the degree of homology between primers and their corresponding *Brassica *sequence.

The amplified sequences were labelled with digoxygenin according the manufacturer's (Roche Diagnostics, Laval, Quebec) instructions and hybridised to colony lifts of a genomic library derived from a *B. rapa Rfp *containing doubled haploid individual, as described [20].

### Sequence analysis

Sequencing and sequence assembly was performed by DNALandmarks (St-Jean-sur-Richelieu) [10] and Genome Quebec (Montreal) using the Applied Biosystems 3730XL DNA analyzer for capillary sequencing and the Phred/Phrap programs for some of the sequence assembly. Additional sequences obtained via shotgun sequencing were assembled using **CodonCode Aligner v.1.3.4 **[37]. Sequences were analysed using **ORF finder **[38] to detect ORFs, **Genscan **[39] to detect promotor regions, introns/exons and polyA signals, and Augustus to detect ORFs, intons and exons [40]. **Blast **and **Blast2Sequences **[41] were used for data mining from nucleotide and protein databases and for aligning pairs of sequences. Tree building was performed with **TreeTop **[42] using the Blosum62 matrix and phylip tree building software. Multiple sequence alignments were performed using ClustalW online [43]. Protein comparisons were based on the ClustalW aligments whenever possible. The output was shaded using Boxshade online [44]. Subcellular targeting predictions were made using online programs Mitoprot [45] and Predotar [46]. The sequences of clones P2, P2-9 and IJC2 have been deposited in GenBank and are listed under accession numbers EF584011, EF584012 and EF584013 respectively.

Criteria for choosing pairs of duplicated genes included online annotation mapping [47,48] and BLAST sequence alignment of the entire CDS and protein sequences with a cutoff expect value of 1e-20 and bit score of >100.

*Arabidopsis *annotation is as per the Munich Information Center for Protein Sequences (MIPS) [49]. In some cases, prediction of PPR domains was made using TPRpred [50].

## Authors' contributions

RG selected clones, performed sequence assembly and annotation, performed the alignment analysis, calculated the Ka and Ks values, and created a draft of the manuscript. GGB conceived of and supervised the work and participated in the drafting and editing of the manuscript.

## Supplementary Material

Additional file 1 Alignment of protein coding sequences of PPR encoding restorer of fertility genes with multiple PPR genes of the 4.3 and 23 megabase regions. This alignment emphasizes the similarity among this group of genes, all of which are from the P subfamily of PPR genes. The asterisk (*) indicates a sequence insertion present only in *Rfo*. Click here for file

## Figures and Tables

**Table 1:** Conservation of genes within two regions of *Arabidopsis *chromosome 1.

Location	4.3 Mb region^2^	23 Mb region^3^
Number of genes/ORFs	19	18
PPR-encoding genes	3	4
Conserved genes	8	8
Hypothetical proteins	2 (+ 1 transposon)	1
% conserved genes^1 ^(all, discounting PPRs)	50	57

^1 ^Percentage of conservation is calculated based on number of genes conserved out of the total number of genes, including hypothetical proteins but not including PPR genes. For example, in the 4.3 Mb region, this is 9 conserved genes out of (19 genes/ORFs -3 PPR-encoding genes) or 9 ÷ 16 = 56%. PPR genes are discounted due to the difficulty in concretely matching PPRs by sequence comparison, and also because of the variability in number and location of PPR genes. Hypothetical genes are annotated according to the *Arabidopsis *annotation according to TAIR.

^2 ^The 4.3 Mb region includes genes DDF1 through At1g12780.

^3 ^The 23 Mb region includes genes DDF2 through At1g63180.

**Table 2:** Relatedness of genes of the 4.3 and 23 Mb syntenic regions of *Arabidopsis *in pairwise comparisons of coding regions at the nucleotide and protein levels.

4.3 Mb region locus	Coding function	23 Mb region locus	Coding function	% nucleotide identity^1^	% amino acid identity/similarity^1^
At1g12610 (DDF1)	DREB subfamily Transcription factor	At1g63030 (DDF2)	DREB subfamily Transcription factor	79%	77%/86%
At1g12630	DREB subfamily Transcription factor	At1g63040	DREB subfamily Transcription factor pseudogene	76%	65%/78%
At1g12640	O-acyl transferase protein	At1g63050	O-acyl transferase protein	84%	83%/89%
At1g12710	F-box family protein	At1g63090	F-box family protein	81%	76%/88%
At1g12730	CDC-like protein	At1g63110	CDC-like protein	83%	76%/84%
At1g12750	Rhomboid family protein	At1g63120	Rhomboid family protein	78%	65%/76%
At1g12760	Zinc finger protein	At1g63170	Zinc finger protein	80%	75%/83%
At1g12780	UDP-glucose epimerase	At1g63180	UDP-glucose epimerase	86%	89%/93%

^1 ^identity and similarity were calculated with the sequence alignment program blast2sequences  to compare whole coding regions.

**Table 3:** Annotation and subcellular localization of PPR-encoding proteins from various plants.

	Gene^1^	Subcellular localization^2^
*Brassica*	Similar to At1g12300	Mitochondrial
*Brassica *cosmid P2-9	Similar to At1g12775	None predicted
*Brassica *cosmid IJC2	Similar to At1g12775	None predicted
Radish *Rfo *region	G1	None predicted
*Arabidopsis *chromosome 1, 4.3 Mb region	At1g12620	Mitochondrial
	At1g12700	Mitochondrial
	At1g12775	Mitochondrial
*Arabidopsis *chromosome 1, 23 Mb region	At1g63070	Mitochondrial
	At1g63080	Mitochondrial
	At1g63130	Mitochondrial
	At1g63150	Mitochondrial
P*	At1g31840	None predicted
P	At5g43820	Mitochondrial
E*	At1g77010	Mitochondrial
E	At2g03380	Mitochondrial
E+*	At1g17630	Possibly mitochondrial
E+	At3g13880	Possibly mitochondrial
DYW*	At1g59720	None predicted
DYW	At5g13230	Mitochondrial
PLS*	At1g14470	None predicted
PLS	At4g18520	Possibly mitochondrial

^1 ^All gene entries beginning with "At" are from *Arabidopsis thaliana*.

^2 ^Subcellular localization was predicted by Mitoprot and Predotar online programs.

* Letter codes correspond to subgroups of PPR-encoding genes as defined by Lurin *et al*. (2004)

**Table 4:** Synonymous and nonsynonymous nucleotide substitution in pairwise comparisons of sequences from radish and *Arabidopsis*.

Genes^1^	Synonymous nucleotide substitutions (Ks)	Nonsynonymous nucleotide substitutions (Ka)	Ka:Ks^2^
*Rfo-g24*	43	84	1.95
*Rfo-g27*	48	76	1.58
*g24-g27*	43	93	2.16
*g19*-At1g63640	167	123	0.74
*g21*-At1g63650	111	107	0.96
*g23*-At1g63680	218	88	0.40

^1 ^*Rfo*, g19, g21, g23, g24 and g27 are all genes from radish, while those starting "At" are genes from *Arabidopsis*. The entire protein coding regions were analysed.

^2 ^Where Ka:Ks = 1 there is no selective pressure acting on the sequence; where Ka:Ks < 1 purifying selection, with a minimization of mutations acts on the sequence; where Ka:Ks > 1 diversifying selection favoring amino acid change acts on the sequence.
