# Supplementary material for: Genes encoding pentatricopeptide repeat (PPR) proteins are not conserved in location in plant genomes and may be subject to diversifying selection
Source: BMC Genomics. 2007 May 23;8:130. doi: 10.1186/1471-2164-8-130 (PMC1892557; doi:10.1186/1471-2164-8-130)
Supplement: Additional file 1 — Alignment of protein coding sequences of PPR encoding restorer of fertility genes with multiple PPR genes of the 4.3 and 23 megabase regions. This alignment emphasizes the similarity among this group of genes, all of which are from the P subfamily of PPR genes. The asterisk (*) indicates a sequence insertion present only in Rfo. [file 1471-2164-8-130-S1.pdf]

```
1g12620        1 VARTKQYDLVLDLCKQMELKGIAHNLYTLSIMINCCCRCRKLSLAFSAMGKIIKLGYEPDTVTFSTLINGLCLEGRVSEA
1g12770        1 IAKTKQYELVLALCKQMESKGIAHSIYTLSIMINCFCRCRKLSYAFSTMGKIMKLGYEPDTVIFNTLLNGLCLECRVSEA
1g12700        1 IARTKQFNLVLDFCKQLELNGIAHNIYTLNIMINCFCRCCKTCFAYSVLGKVMKLGYEPDTTTFNTLIKGLFLEG-----
1g63070        1 IAKMNKFDLVISLGEQMQNLGISHNLYTYSIFINYFCRRSQLSLALAILGKMMKLGYGPSIVTLNSLLNGFCHGNRISEA
1g63080        1 IAKMKKFDLVISFGEKMEILGVSHNLYTYNIMINCLCRRSQLSFALAILGKMMKLGYGPSIVTLNSLLNGFCHGNRISEA
1g63130        1 IAKMNKFDLVISLGEQMQNLGISHNLYTYSILINCFCRRSQLSLALAVLAKMMKLGYEPDIVTLNSLLNGFCHGNRISDA
1g63150        1 VAKMNKFELVISLGEQMQTLGISHDLYTYSIFINCFCRRSQLSLALAVLAKMMKLGYEPDIVTLSSLLNGYCHSKRISDA
Rfo            1 VVRMERPDLVISLYQKMERKQIRCDIYSFNILIKCFCSCSKLPFALSTFGKITKLGLHPDVVTFTTLLHGLCVEDRVSEA
Rf-1petunia    1 LVHMKHYSSVVSIFREIHKLRIPVDAFALSTVVNSCCLMHRTDLGFSVLAIHFKKGIPYNEVTFTTLIRGLFAEN-----
Rf-1rice       1 LCKAQAMDKAMEVLNTMVKNGVMPDCMTYNSILHGYCSSGQPKEAIGFLKKMRSDGVEPDVVTYSLLMDYLCKNGRCMEA
consensus      1 iakmkkfdlvisl  qme lgi hnlytysimincfCr sqlslalsvlgkmmklGyepdivtfntLlnglche rvsea

1g12620       81 LELVDRMVEMGHKPTLITLNALVNGLCLNGKVSDAVLLIDRMVET-GFQPNEVTYGPVLKVMCKSGQTALAMELLRKMEE
1g12770       81 LELVDRMVEMGHKPTLITLNTLVNGLCLNGKVSDAVVLIDRMVET-GFQPNEVTYGPVLNVMCKSGQTALAMELLRKMEE
1g12700       76 ------------------------------KVSEAVVLVDRMVEN-GCQPDVVTYNSIVNGICRSGDTSLALDLLRKMEE
1g63070       81 VALVDQMVEMGYQPDTVTFTTLVHGLFQHNKASEAVALVERMVVK-GCQPDLVTYGAVINGLCKRGEPDLALNLLNKMEK
1g63080       81 VALVDQMVEMGYQPDTVTFTTLVHGLFQHNKASEAVALVERMVVK-GCQPDLVTYGAVINGLCKRGEPDLALNLLNKMEK
1g63130       81 VSLVGQMVEMGYQPDSFTFNTLIHGLFRHNRASEAVALVDRMVVK-GCQPDLVTYGIVVNGLCKRGDIDLALSLLKKMEQ
1g63150       81 VALVDQMVEMGYKPDTFTFTTLIHGLFLHNKASEAVALVDQMVQR-GCQPDLVTYGTVVNGLCKRGDIDLALNLLNKMEA
Rfo           81 LDFFHQMFETTCRPNVVTFTTLMNGLCREGRIVEAVALLDRMMED-GLQPTQITYGTIVDGMCKKGDTVSALNLLRKMEE
Rf-1petunia   76 ------------------------------KVKDAVHLFKKLVRENICEPDEVMYGTVMDGLCKKGHTQKAFDLLRLMEQ
Rf-1rice      81 RKIFDSMTKRGLKPEITTYGTLLQGYATKGALVEMHGLLDLMVRN-GIHPDHYVFSILICAYAKQGKVDQAMLVFSKMRQ
consensus     81 l lvdqmvemg kpd itf tlv gl  h kvseavaLvdrmv   gcqPdvvtyg vlnglckrGdtdlAl llrkMe

1g12620      160 R-KIKLDAVKYSIIIDGLCKDGSLDNAFNLFNEMEIKGFKADIIIYTTLIRGFCYAGRWDDGAKLLRDMIKRKITPDVVA
1g12770      160 R-NIKLDAVKYSIIIDGLCKDGSLDNAFNLFNEMEIKGFKADIITYNTLIGGFCNAGRWDDGAKLLRDMIKRKISPNVVT
1g12700      125 R-NVKADVFTYSTIIDSLCRDGCIDAAISLFKEMETKGIKSSVVTYNSLVRGLCKAGKWNDGALLLKDMVSREIVPNVIT
1g63070      160 G-KIEADVVIYNTIIDGLCKYKHMDDAFDLFNKMETKGIKPDVFTYNPLISCLCNYGRWSDASRLLSDMLEKNINPDLVF
1g63080      160 G-KIEADVVIYSTVIDSLCKYRHVDDALNLFTEMDNKGIRPDVFTYSSLISCLCNYGRWSDASRLLSDMLERKINPNVVT
1g63130      160 G-KIEPGVVIYNTIIDALCNYKNVNDALNLFTEMDNKGIRPNVVTYNSLIRCLCNYGRWSDASRLLSDMIERKINPNVVT
1g63150      160 A-RIKANVVIFNTIIDSLCKYRHVEVAVDLFTEMETKGIRPNVVTYNSLINCLCNYGRWSDASRLLSNMLEKKINPNVVT
Rfo          160 VSHIIPNVVIYSAIIDSLCKDGRHSDAQNLFTEMQEKGIFPDLFTYNSMIVGFCSSGRWSDAEQLLQEMLERKISPDVVT
Rf-1petunia  126 G-ITKPDTCIYNIVIDAFCKDGMLDGATSLLNEMKQKNIPPDIITYTSLIDGLGKLSQWEKVRTLFLEMIHLNIYPDVCT
Rf-1rice     160 Q-GLNPNAVTYGAVIGILCKSGRVEDAMLYFEQMIDEGLSPGNIVYNSLIHGLCTCNKWERAEELILEMLDRGICLNTIF
consensus    161 g kik dvviystiIdglCkdghlddAinlf eMe kgikpdvitYnsli glcn grWsda kLl dMlerkI pnvvt

1g12620      239 FSALIDCFVKEGKLREAEELHKEMIQRGISPDTVTYTSLIDGFCKENQLDKANHMLDLMVSKGCGPNIRTFNILINGYCK
1g12770      239 FSVLIDSFVKEGKLREADQLLKEMMQRGIAPNTITYNSLIDGFCKENRLEEAIQMVDLMISKGCDPDIMTFNILINGYCK
1g12700      204 FNVLLDVFVKEGKLQEANELYKEMITRGISPNIITYNTLMDGYCMQNRLSEANNMLDLMVRNKCSPDIVTFTSLIKGYCM
1g63070      239 FNALIDAFVKEGKLVEAEKLYDEMVK----------------------------------SKHCFPDVVAYNTLIKGFCK
1g63080      239 FNSLIDAFAKEGKLIEAEKLFDEMIQRSIDPNIVTYNSLINGFCMHDRLDEAQQIFTLMVSKDCLPDVVTYNTLINGFCK
1g63130      239 FSALIDAFVKEGKLVEAEKLYDEMIKRSIDPDIFTYSSLINGFCMHDRLDEAKHMFELMISKDCFPNVVTYNTLIKGFCK
1g63150      239 FNALIDAFFKEGKLVEAEKLHEEMIQRSIDPDTITYNLLINGFCMHNRLDEAKQMFKFMVSKDCLPNIQTYNTLINGFCK
Rfo          240 YNALINAFVKEGKFFEAEELYDEMLPRGIIPNTITYSSMIDGFCKQNRLDAAEHMFYLMATKGCSPNLITFNTLIDGYCG
Rf-1petunia  205 FNSVIDGLCKEGKVEDAEEIMTYMIEKGVEPNEITYNVVMDGYCLRGQMGRARRIFDSMIDKGIEPDIISYTALINGYVE
Rf-1rice     239 FNSIIDSHCKEGRVIESEKLFELMVRIGVKPNVITYNTLINGYCLAGKMDEAMKLLSGMVSVGLKPNTVTYSTLINGYCK
consensus    241 fnalidafvKEGklveaeklydeMi rgi pn itynslidgfcm nrldea hmfdlmvskgc P ivtyntLInGyck

1g12620      319 ANLIDDGLELFRKMSLRGVVADTVTYNTLIQGFCELGKLEVAKELFQEMVSRRVRPDIVSYKILLDGLCDNGEPEKALEI
1g12770      319 ANRIDDGLELFREMSLRGVIANTVTYNTLVQGFCQSGKLEVAKKLFQEMVSRRVRPDIVSYKILLDGLCDNGELEKALEI
1g12700      284 VKRVDDGMKVFRNISKRGLVANAVTYSILVQGFCQSGKIKLAEELFQEMVSHGVLPDVMTYGILLDGLCDNGKLEKALEI
1g63070      285 YKRVEEGMEVFREMSQRGLVGNTVTYTTLIHGFFQARDCDNAQMVFKQMVSDGVHPDIMTYNILLDGLCNNGNVETALVV
1g63080      319 AKKVVDGMELFRDMSRRGLVGNTVTYTTLIHGFFQASDCDNAQMVFKQMVSDGVHPNIMTYNTLLDGLCKNGKLEKAMVV
1g63130      319 AKRVDEGMELFREMSQRGLVGNTVTYTTLIHGFFQARECDNAQIVFKQMVSDGVLPDIMTYSILLDGLCNNGKVETALVV
1g63150      319 CKRVEDGVELFREMSQRGLVGNTVTYTTIIQGFFQAGDCDSAQMVFKQMVSNRVPTDIMTYSILLHGLCSYGKLDTALVI
Rfo          320 AKRIDDGMELLHEMTETGLVADTTTYNTLIHGFYLVGDLNAALDLLQEMISSGLCPDIVTCDTLLDGLCDNGKLKDALEM
Rf-1petunia  285 KKKMDKAMQLFREISQNGLKPSIVTCSVLLRGLFEVGRTECAKIFFDEMQAAGHIPNLYTHCTLLGGYFKNGLVEEAMSH
Rf-1rice     319 ISRMEDALVLFKEMESSGVSPDIITYNIILQGLFQTRRTAAAKELYVRITESGTQIELSTYNIILHGLCKNKLTDDALQM
consensus    321 akrvddgmelfrems rGlvantvTystliqGffq g  e A mlf emvs gv pdimty ilLdGlc ngkle Al i

1g12620      399 FEKIEKSK-----------MELDIGIYNIIIHGMCNASKVDDAWDLFCSLPLKGVKPDVKTYNIMIGGLCKKGSLSEADL
1g12770      399 FGKIEKSK-----------MELDIGIYMIIIHGMCNASKVDDAWDLFCSLPLKGVKLDARAYNIMISELCRKDSLSKADI
1g12700      364 FEDLQKSK-----------MDLGIVMYTTIIEGMCKGGKVEDAWNLFCSLPCKGVKPNVMTYTVMISGLCKKGSLSEANI
1g63070      365 FEYMQKRD-----------MKLDIVTYTTMIEALCKAGKVEDGWDLFCSLSLKGVKPNVVTYTTMMSGFCRKGLKEEADA
1g63080      399 FEYLQKSK-----------MEPDIYTYNIMSEGMCKAGKVEDGWDLFCSLSLKGVKPDVIAYNTMISGFCKKGLKEEAYT
1g63130      399 FEYLQRSK-----------MEPDIYTYNIMIEGMCKAGKVEDGWDLFCSLSLKGVKPNVVTYTTMMSGFCRKGLKEEADA
1g63150      399 FKYLQKSE-----------MELNIFIYNTMIEGMCKAGKVGEAWDLFCSLSIK---PDVVTYNTMISGLCSKRLLQEADD
Rfo          400 FKVMQKSKKDLDASHPFNGVEPDVQTYNILISGLINEGKFLEAEELYEEMPHRGIVPDTITYSSMIDGLCKQSRLDEATQ
Rf-1petunia  365 FHKLERRR-----------EDTNIQIYTAVINGLCKNGKLDKAHATFEKLPLIGLHPDVITYTAMISGYCQEGLLDEAKD
Rf-1rice     399 FQNLCLMD-----------LKLEARTFNIMIDALLKVGRNDEAKDLFVAFSSNGLVPNYWTYRLMAENIIGQGLLEELDQ
consensus    401 Fe lqksk           meldi tynimiegmckagkvddawdlfcsl lkgvkpdvmtYttMisglckkglleead

1g12620      468 LFRKMEEDGHSPNGCTYNILIRAHLGEGDATKSAKLIEEIKRCGFSVDAST-VKMVVDML
1g12770      468 LFRKMTEEGHAPDELTYNILIRAHLGDDDATTAAELIEEMKSSGFPADVST-VKMVINML
1g12700      433 LLRKMEEDGNAPNDCTYNTLIRAHLRDGDLTASAKLIEEMKSCGFSADASS-IKMVIDML
1g63070      434 LFVEMKEDGPLPNSGTYNTLIRARLRDGDEAASAELIKEMRSCGFAGDAST-FGLVTNML
1g63080      468 LFIKMKEDGPLPDSGTYNTLIRAHLRDGDKAASAELIKEMRSCRFAGDAST-YGLVTDML
1g63130      468 LFREMKEEGPLPDSGTYNTLIRAHLRDGDKAASAELIREMRSCRFVGDAST-IGLVTNML
1g63150      465 LFRKMKEDGTLPNSGTYNTLIRANLRDCDRAASAELIKEMRSSGFVGDAST-ISLVTNML
Rfo          480 MFDSMGSKSFSPNVVTFTTLINGYCKAGRVDDGLELFCEMGRRGIVANAITYITLICGFR
Rf-1petunia  434 MLRKMEDNGCLPDNRTYNVIVRGFFRSSKVSEMKAFLKEIAGKSFSFEAAT-VELLMDII
Rf-1rice     468 LFLSMEDNGCTVDSGMLNFIVRELLQRGEITRAGTYLSMIDEKHFSLEAST-ASLFIDLL
consensus    481 lfrkM edg lp sgtyntlirahlrdgd tasaelikemkscgf adast v lvvdml
```
